# EHMTI-0280. Cortical excitability changes in chronic migraine vs episodic migraine: evidence by sound-induced flash illusions

**DOI:** 10.1186/1129-2377-15-S1-E5

**Published:** 2014-09-18

**Authors:** F Brighina, N Bolognini, G Vallar, G Cosentino, S Maccora, P Paladino, S Indovino, R Baschi, B Fierro

**Affiliations:** 1 Dept of Experimental Biomedicine and Clinical Neuroscience (Bionec), University of Palermo Italy, Palermo, Italy; 2 Dept. of Psychology, University of Milano.Bicocca Italy, Milano, Italy; 3 Dept of Experimental Biomedicine and Clinical Neuroscience (Bionec), University of Palermo Italy, Palermo, Italy

## Introduction

Sound-induced flash illusions(SIFI) permit to evaluate cross-modal audio-visual perception. When one flash is accompanied by two beeps, it is perceived as two flashes('fission'illusion); a 'fusion' illusion occurs when a single beep causes the fusion of a double flash stimulus. SIFI strictly depends on cortical excitability: healthy controls perceive less illusions by increasing visual cortex excitability through anodal tDCS [1].

## Aim

to evaluate if, due to cortical hyperexcitability, differences in SIFI occur in migraine and further changes can be found across migraine cycle, migraine chronification an drug overuse.

## Methods

we enrolled 64 patients with episodic migraine, 32 with-(MWA) and 32 without-aura(MWO) (42 F, mean age 32,3±16yrs), 44 patients with chronic migraine with medication overuse headache (36 F, mean age 39.2±12.2), and 20 healthy controls (13 F, mean age 38±18). All underwent a paradigm for SIFI induction where had to report the number of flashes seen. 13 of MWO and 12 out of MWA were examined in both ictal and interictal phase

## Results

all migraine groups showed significantly less SIFI than controls(p<.0001); illusions are more reduced in in chronic migraine and particularly in those overusing triptans(p<.05).

## Conclusions

results point to a condition of visual cortical hyperresponsivity in patients with chronic migraine in analogy to what observed in episodic patients expecially during ictal phase. This is in agreement with the view of chronic migraine as a 'never ending attack'. The greater effect showed in triptan overuser can follow to down-regulation of 5HT1 receptors.

No conflict of interest.

## References

[B1] Bolognini N Neuropsychologia 2011 49 231 7 10.1016/j.neuropsychologia.2010.11.015 21094177

